# Hemophagocytic lymphohistiocytosis as the initial manifestation of Epstein–Barr virus-related T/NK-cell lymphoproliferative disorders in a pediatric patient: a case report and literature review

**DOI:** 10.3389/fped.2025.1662074

**Published:** 2025-11-11

**Authors:** Lianwei Ma, Xue Xing, Yonge Liu, Wei Jiang

**Affiliations:** Department of Clinical Laboratory, The Second Affiliated Hospital of Dalian Medical University, Liaoning, China

**Keywords:** hemophagocytic lymphohistiocytosis (HLH), Epstein–Barr virus (EBV), aggressive natural killer cell leukemia (ANKL), lymphoproliferative disorders (LPDs), pediatric, *DDX3X* mutation, *KMT2D* mutation

## Abstract

Epstein–Barr virus-associated T/Natural Killer-cell lymphoproliferative disorders (EBV-T/NK-LPDs) are rare diseases that predominantly affect children and young adults. We report a case of a 12-year-old child who initially presented with EBV-associated hemophagocytic lymphohistiocytosis (HLH) and later progressed to aggressive NK-cell leukemia (ANKL). Both EBV-HLH and ANKL belong to the spectrum of EBV-T/NK-LPDs, which also includes chronic active EBV infection (CAEBV) of T-cell and NK-cell types, systemic EBV-positive T-cell lymphoma of childhood, extranodal NK/T-cell lymphoma (ENKTL), and primary EBV-positive nodal T/NK-cell lymphoma. The patient initially presented with recurrent high fever, pancytopenia, hepatosplenomegaly, hypertriglyceridemia, and hypofibrinogenemia with a significantly elevated EBV-DNA load. Bone marrow examination indicated a few histiocytes and hemophagocytic cells. Subsequent increases in ferritin and soluble CD25 (sCD25) levels were further consistent with the diagnosis of EBV-HLH. No lymphadenopathy or nasal primary lesion was identified. Further BM flow cytometry and BM cell morphology examination indicated abnormal NK-cell infiltration, leading to the exclusion of ENKTL, primary EBV-positive nodal T/NK-cell lymphoma, and systemic EBV-positive T-cell lymphoma of childhood. A diagnosis of ANKL was highly suspected. This case highlights that when EBV infection triggers a series of complex EBV-T/NK-LPDs occurring sequentially or simultaneously, the differential diagnosis and treatment become difficult, which can easily lead to delays in diagnosis and treatment. Developing optimized diagnostic algorithms and evidence-based treatment strategies is essential to improve outcomes of patients.

## 1 Introduction

Epstein–Barr virus (EBV) is a ubiquitous herpesvirus that infects more than 90% of the world's population and is linked to many malignancies and pathological states (1, 2). EBV primarily exhibits tropism for B cells but can also infect natural killer (NK) cells, T cells, and epithelial cells (3). Children under six years infected with EBV are mostly asymptomatic or exhibit non-specific symptoms, and approximately 50% of adolescents infected with this virus develop infectious mononucleosis (4). In a few cases, the finely controlled balance between persistent active EBV infection and the host immune response is disrupted, leading to various EBV-associated lymphoproliferative disorders (LPDs) of B, T, or NK cells (5–7). Among them, EBV-associated T-/NK -cell LPDs (EBV-T/NK-LPDs) are rare diseases that predominantly affect children and young adults and are associated with high mortality (8). EBV-T/NK-LPD are more common among Asians and indigenous peoples in Central and South America compared to the Western populations (9).

EBV-T/NK-LPDs represent a broad clinicopathological spectrum ranging from indolent, self-limiting, and localized conditions to highly aggressive lymphomas (6, 7). According to the World Health Organization classification of lymphoid neoplasms (2022) (10) and previous reports (5, 6, 9, 11), EBV-T/NK-LPDs involve non-neoplastic lesions, such as EBV-associated haemophagocytic lymphohistiocytosis (EBV-HLH), chronic conditions with variable outcomes, such as chronic active EBV infections (CAEBV) of T- and NK-cell type, and malignant diseases, such as systemic EBV-positive T-cell lymphoma of childhood, aggressive NK-cell leukemia (ANKL), extranodal NK/T-cell lymphoma (ENKTL), and primary EBV-positive nodal T/NK-cell lymphoma. Due to the rarity, broad clinicopathological spectrum, and significant morphological and immunophenotypic overlap of EBV-T/NK-LPDs, their diagnosis and precise classification often pose a challenge to clinicians.

Herein, we report a case of a 12-year-old child with ANKL who initially presented with hemophagocytic lymphohistiocytosis (HLH) following EBV infection. The findings presented here will help increase our understanding of this rare disease.

## 2 Case description

A 12-year-old girl was admitted to our hospital with a 15-day history of intermittent fever. Her temperature peaked at 39°C and was accompanied by chills, dizziness, and cold sensation. Administering oral antipyretics and physical cooling normalized her body temperature.

One week prior to admission, a chest radiograph revealed patchy ground-glass opacities and high-density nodular shadows in both lungs. Blood tests revealed anemia (hemoglobin, 114 g/L), leucopenia [white blood cells (WBCs), 3.05 × 10^9^/L], and an elevated lymphocyte percentage (L%, 55.8%). An abdominal ultrasound revealed hepatosplenomegaly. The patient was treated with azithromycin, ceftazidime, and methylprednisolone for a few days. However, during treatment, the patient developed thrombocytopenia (platelet count, 119 × 10^9^/L) with elevated levels of alanine transaminase (ALT, 255 U/L) and aspartate aminotransferase (AST, 242 U/L). Results of the laboratory examination prior to admission are described in Table 1.

**Table 1:** Laboratory data at the following time points: the day prior to admission, first admission, and second admission.

Laboratory data	Value at the day prior to admission	Value at first admission	Value at second admission	Reference range
Hemoglobin (g/L)	111	103	95	115–150
Platelets/L	77 × 10^9^	73 × 10^9^	19 × 10^9^	125–350 × 10^9^
White blood cells/L	2.8 × 10^9^	2.25 × 10^9^	1.44 × 10^9^	3.5–9.5 × 10^9^
Neutrophils/L	1.23 × 10^9^	0.80 × 10^9^	0.35 × 10^9^	1.8–6.3 × 10^9^
Activated partial thromboplastin time (s)	NA	47.5	44.5	26–43
Thrombin time (s)	NA	21.5	21.2	14–21
Fibrinogen (g/L)	1.51	1.64	1.66	2–4
Triglyceride (mmol/L)	2.28	2.95	1.67	0.22–1.7
Alanine aminotransferase (U/L)	186.00	124.90	444.65	7–40
Aspartate aminotransferase (U/L)	117.00	82.61	219.53	13–35
Serum albumin (g/L)	NA	31.38	31.1	40–55
Total bile acid (μmol/L)	NA	15.72	18.74	0–10
Prealbumin (mg/L)	NA	155.39	134.93	250–400
Lactate dehydrogenase (U/L)	NA	391.86	437.53	120–250
Epstein–Barr virus DNA (IU/ml, serum sample)	NA	31,783.3	89,766.7	<1,667
Ferritin (μg/L)	NA	129.71	600.09	10–291
Serum ferrium (μmol/L)	NA	5.08	NA	7.8–32.2
Percent iron saturation (%)	NA	7.93	NA	33–55
Unsaturated iron binding capacity (μmol/L)	NA	59.02	NA	25–50.1
Direct Coombs test	NA	+	NA	−
NK-cell perforin (%)	NA	68.3	NA	81–100
NK-cell granzyme B (%)	NA	92.1	NA	77–100
CD8 ^+^ T cell perforin (%)	NA	74.5	NA	2–100
CD8 ^+^ T cell granzyme B (%)	NA	26.6	NA	6–100
IgE (IU/ml)	NA	6,400	3,590	0–100
IgG (g/L)	NA	11.2	9.09	7–16
IgA (g/L)	NA	2.11	1.57	0.7–4.0
IgM (g/L)	NA	0.35	0.46	0.4–2.3
Lymphocyte Count (cells/μl)	NA	1,253	NA	1,230–3,100
Total T Lymphocytes (CD3^+^, %)	NA	54.87	NA	60–84
Total T Lymphocytes Count (cells/μl)	NA	812	NA	955–2,860
Helper/Inducer T Lymphocytes (CD4^+^, %)	NA	37.34	NA	36–55
Helper/inducer T Lymphocytes Count (cells/μl)	NA	439	NA	680–1,440
Suppressor/Cytotoxic T Lymphocytes (CD8^+^, %)	NA	24.16	NA	20–35
Suppressor/Cytotoxic T Lymphocytes Count (cells/μl)	NA	284	NA	360–1,250
CD4 + CD8+ Double Positive Cells (%)	NA	1.78	NA	1–5
Helper/Suppressor Ratio (CD4+/CD8+, ratio)	NA	1.55	NA	1.4–2.0
Total B Lymphocytes (%)	NA	11.3	NA	5–20
B Lymphocytes Count (cells/μl)	NA	150	NA	90–580
NK Cells (%)	NA	23.26	NA	8–28
NK Cells Count (cells/μl)	NA	309	NA	150–900
NK-T Cells (%)	NA	1.44	NA	1–5
γ δ T Cells (%)	NA	5.25	NA	1–5
Interleukin-1β (pg/ml)	NA	8.18	NA	0–5
soluble Interleukin-2 receptor (U/ml)	NA	2,134	>7,500	223–710
Interleukin-6 (pg/ml)	NA	6.44	16.8	0–3.4
Interleukin-8 (pg/ml)	NA	6.67		0–62
Interleukin-10 (pg/ml)	NA	27.7	76.16	0–9.1
Tumor necrosis factors-α (pg/ml)	NA	27.5	3.33	0–8.1
Complement C3 (g/L)	NA	1.020	1.420	0.9–1.8
Complement C4 (g/L)	NA	0.389	0.597	0.1–0.4

Data are presented as the actual single measurements from the individual patient.

The patient was admitted to our hospital due to poor response to the treatment and a worsening of symptoms. She had undergone bilateral tonsillectomy in 2015 and had no history of other diseases; her family history was also unremarkable. On physical examination, hepatosplenomegaly was noted. The superficial lymph nodes in the body were neither palpable nor swollen. Complete results of the laboratory examination after the first admission are described in Table 1. The patient presented with pancytopenia, and the Wright-Giemsa-stained peripheral blood smear revealed no obvious abnormality. Further examination of the bone marrow (BM) cell morphology indicated pancytopenia with iron deficiency BM and a few histiocytes and hemophagocytic cells. She experienced a decrease in fibrinogen (FIB), alongside an increase in triglyceride (TG) levels. The increase in EBV-DNA load (31,783.3 IU/ml, 1 IU/ml = 0.6 copies/ml)in the patient's serum suggested the presence of EBV infection. Serological tests for immunoglobulin (Ig)M antibodies against influenza A, influenza B, parainfluenza virus, adenovirus, respiratory syncytial virus, Mycoplasma pneumoniae, Chlamydia pneumoniae, and Legionella pneumophila were negative. Nucleic acid tests for Cytomegalovirus (CMV) and COVID-19 were negative.

Immunologic evaluation: During the initial admission to our hospital, immunologic evaluation revealed decreased perforin expression in NK cells, whereas granzyme B levels in NK cells were within normal limits. Both perforin and granzyme B expression in CD8^+^ T cells were normal. Serum immunoglobulin assays showed a significant elevation in IgE (6,400 IU/ml) with other immunoglobulin levels within normal ranges (Table 1). Upon readmission (35 days after the first admission), IgE levels remained markedly elevated (3,590 IU/ml), though decreased compared to the initial value, while other immunoglobulins remained normal (Table 1). Lymphocyte subset analysis at the first admission demonstrated an elevated percentage of γδ T cells (5.25%), along with decreased absolute counts of total T lymphocytes (CD3^+^: 812 cells/μl), helper/inducer T lymphocytes (CD3^+^CD4^+^: 439 cells/μl), and suppressor/cytotoxic T lymphocytes (CD3^+^CD8^+^: 284 cells/μl). Other subsets, including NK cells (percentage: 23.26%; absolute count: 309 cells/μl), were within normal ranges (Table 1). Furthermore, the patient exhibited elevated levels of various inflammatory factors, indicative of a cytokine storm (Table 1). The soluble interleukin-2 receptor (sIL-2R/sCD25) level was significantly elevated at 2,134 U/ml during the first admission and increased further to >7,500 U/ml upon readmission. Complement C3 and C4 levels were normal initially, but C4 was slightly elevated (0.597 g/L) at the second admission (Table 1). Autoantibody profiling, including tests for antinuclear antibody (ANA), rheumatoid factor (RF), anti-neutrophil cytoplasmic antibodies (ANCA), extractable nuclear antigen antibodies (ENA), and anti-double-stranded DNA antibody, was consistently negative. A pulmonary computed tomography (CT) scan indicated pneumonia, accompanied by hepatosplenomegaly (liver, 9.2 cm; spleen, 9.1 cm below the costal margin). The patient received polyene phosphatidylcholine [465 mg, intravenous [iv] drip, once daily [qd]], cefuroxime [2 g, iv drip, twice daily (q12 h)], azithromycin (0.5 g, iv drip, qd), methylprednisolone (30 mg, iv drip, qd) and ganciclovir (0.25 g, IV, qd), resulting in five days of normal temperature. However, on day 8, she developed a fever of 37.6°C, and the chest CT revealed worsening pulmonary lesions, prompting referral to a higher-level hospital for further management.

The patient was transferred to the superior hospital. EBV serologic tests revealed positive results for EBV-EA-IgG, EBV-NA-IgG, and EBV-VCA-IgG and a negative result for EBV-VCA-IgM after admission (Supplementary Table 1), and ganciclovir was administered. On the fourth day of admission, the soluble CD25 (sCD25) level was 20,404 pg/ml. Methylprednisolone 60 mg was administered intravenously every 12 h to suppress inflammatory responses. The EBV infection lymphocyte subgroups were as follows: CD4^+^ T cells, 1.7 × 10^5^; CD8^+^ T cells, 4.4 × 10^5^; B cells, 1.1 × 10^5^; and NK/NKT cells, 3.5 × 10^6^ (EBV-DNA copies per 1 million cells) (Supplementary Table 1). Flow cytometry analysis of the BM did not reveal any significant abnormalities. Both the BM cytology examination and flow cytometry showed no evidence of malignancy. The patient was definitively diagnosed with hemophagocytic lymphohistiocytosis (HLH), prompting treatment with etoposide (VP16) and dexamethasone. The HLH-2004 criteria (12) and this patient's specific fulfillment of them, with the corresponding timeline, are shown in Supplementary Table 2. The HScore was determined for the patient's initial presentation and subsequent assessment at the superior hospital via the publicly available HScore online system (http://saintantoine.aphp.fr/score/) (13). An identical score of 223 was obtained for both time points, corresponding to a 96.88% probability of HLH. The best cutoff value for HScore was 169, corresponding to a sensitivity of 93%, a specificity of 86%, and accurate classification of 90% of the patients (13). On the eleventh day of admission, the patient was discharged after stabilization and instructed to take oral dexamethasone. Ten days later, she was re-admitted due to a fever (38.4°C) occurring twice daily, mainly in the mornings and evenings, which was relieved by meloxicam. The last BM flow cytometry indicated the presence of 33.7% heterotypic NK lymphocytes, and the sCD25 level was increased to 44,560 pg/ml. BM cell morphology examination revealed 1.6% hemophagocytic cells. T/NK-cell lymphoma was suspected, and NK-cell lymphoma was yet to be excluded.

The child was re-admitted to our hospital for the second time at the request of her family members. She was progressively worsening. The superficial lymph nodes were not palpable or swollen, and the liver and spleen were palpable 2 cm below the costal margin. The laboratory examination results after the second admission are described in Table 1. The Wright-Giemsa-stained peripheral blood smear revealed the presence of 6% neutrophilic myelocytes, 5% neutrophilic metamyelocytes, 20% neutrophils, 56% lymphocytes, 1% monocytes, and 12% atypical lymphocytes. The elevated proportion of abnormal lymphocytes suggests an aggressive tumor phenotype. A diagnosis of NK-cell lymphoma leukemia was reached at the Institute of Hematology and Blood Diseases on the fifth day.

**Figure 1:**
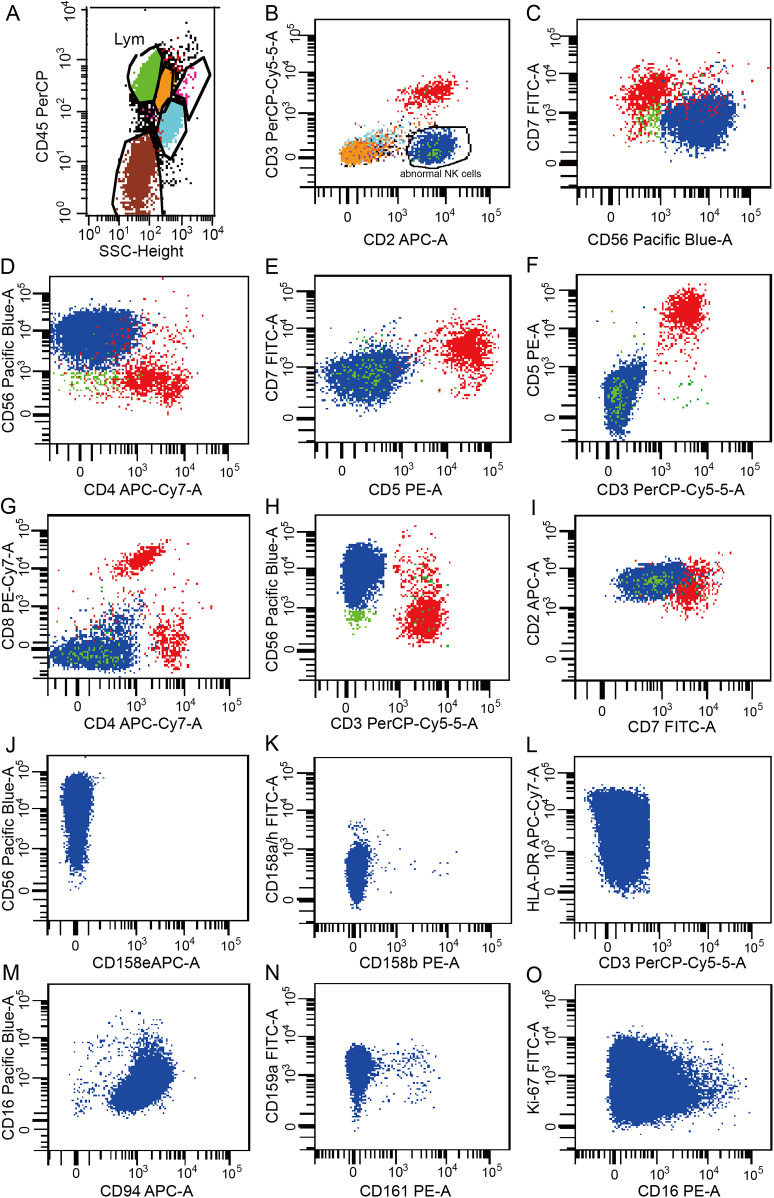
Results of the flow cytometry analysis of peripheral blood. **(A)** Lymphocytes accounted for 60.95% of the nucleated cells, representing an increased proportion. **(B)** Abnormal phenotypic NK cells were observed, accounting for 51.1% of the nucleated cells. **(C–N)** CD2, CD56, HLA-DR (partial), CD94, CD159a and CD16 were expressed. CD3, CD7, CD4, CD8, CD5, CD158e, CD158b, CD158a/h, and CD161 were not expressed. **(O)** Ki-67 expression was observed in 25.2% of the cells. Data are presented as the actual single measurements from the individual patient.

**Figure 2:**
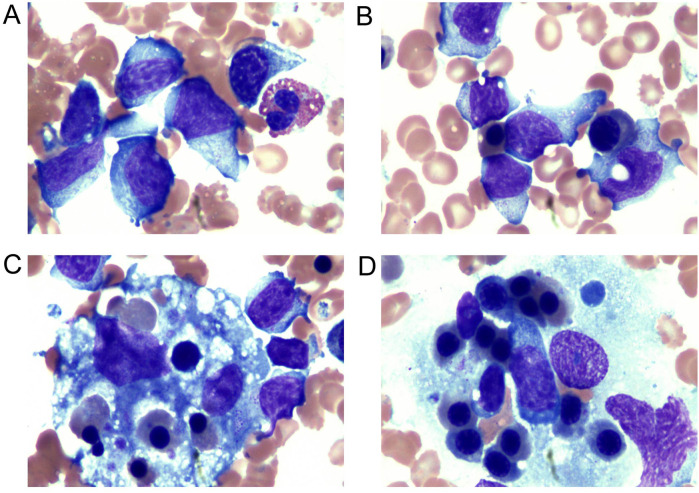
Examination of the bone marrow cell morphology. **(A,B)** NK cells: the cell volume was large, the cell body was irregular, the nucleus was irregular, round, or oval, and some presented with depression and distortion. The nuclear chromatin was partially loose and partially concentrated. The cytoplasm was abundant and blue; small red particles were seen in some areas. **(C,D)** Bone marrow biopsy showed histiocytes actively phagocytosing blood cells. Vacuoles and fragments of phagocytosed nucleated cells and platelets could be seen in the cytoplasm.

**Figure 3:**
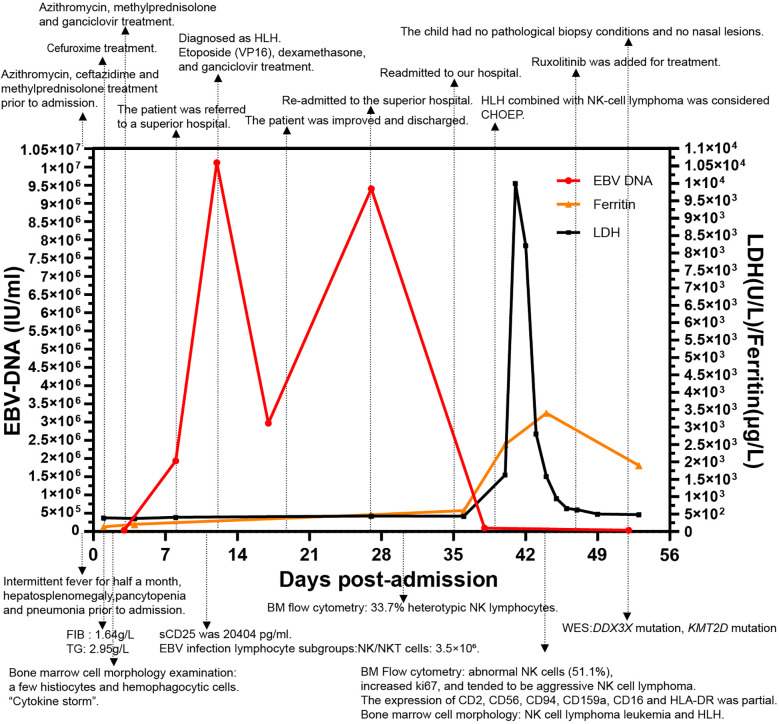
The changes in EBV-DNA levels, inflammatory markers during the disease course, and the progression timeline of the patient. The timeline outlines the different events and disease milestones during treatment. Key therapeutic events and critical incidents are marked on the graph, while notable laboratory findings are described below the figure. Data are presented as the actual single measurements from the individual patient.

The ferritin level had increased to 2,500 μg/L on the sixth day of the second admission. The patient presented with trilineage blood cytopenia, hyperlipidemia, low FIB levels (1.05 g/L), and significantly increased sIL-2R; consequently, a diagnosis of HLH with NK-cell lymphoma was considered. Nasal endoscopy revealed mild mucosal congestion, localized ulceration, and scant bloody secretions. The nasal septum was midline without significant deviation. No masses or neoplastic lesions were observed in either nasal cavity or the nasopharynx. No nasal lesions were detected; ENKTL (nasal type) is currently not suspected. Cyclophosphamide, doxorubicin, vincristine, etoposide, and prednisolone (CHOEP) were used to treat the primary disease and control the progression of HLH. The definitive diagnosis was secured primarily through immunophenotypic profiling via flow cytometry performed on a bone marrow aspirate, in conjunction with a morphological assessment by bone marrow cytology. Flow cytometry (Figure 1) identified a population of abnormal NK cells in the bone marrow (51.1%), exhibiting a phenotype positive for CD2, CD56, and CD16, negative for CD3, cCD3, CD7, CD4, and CD8, consistent with an NK-cell origin. Elevated Ki67 levels were observed. Flow cytometry supporting the diagnosis of aggressive NK-cell lymphoma. CD2, CD56, CD94, CD159a, CD 16 and HLA-DR (partially) were expressed (Figure 1). The results of the BM cell morphology examination (Figure 2) on day 10 of re-admission revealed prominent hemophagocytosis and diffuse infiltration by abnormal lymphocytes characterized by large size, irregular shape, abundant and blue cytoplasm, and small red particles were seen in some areas. This finding is consistent with the flow cytometry results. These findings collectively confirm the final diagnosis of EBV-associated ANKL complicated by hemophagocytic syndrome. On day 13, the patient's blood coagulation profile was normal, and liver function tests showed improvement. However, ruxolitinib was added to the treatment due to recurrent fever. On the 21st day of re-admission, she developed a fever again; a blood routine examination showed no lymphocytes or monocytes. The patient was in a serious and complex condition. Following chemotherapy, the patient was transferred to a tertiary hospital for consideration of allogeneic hematopoietic stem cell transplantation. Unfortunately, no further follow-up data regarding transplant status or clinical outcome could be obtained after the transfer, and the patient was lost to long-term follow-up. The timeline of treatment and key events is shown in Figure 3 and Supplementary Table 1 presents the patient's EBV-related serological and virological test results, along with the corresponding timeline and clinical symptoms. Supplementary Table 3, the Clinical Course Summary Table, presents the main clinical features of the patient, key laboratory data, diagnostic tests, treatment information, as well as considerations for diagnosis and differential diagnosis.

Whole-exome sequencing (WES) was performed on DNA extracted from 2 ml of ethylenediaminetetraacetic acid (EDTA) anticoagulated peripheral whole blood, which was considered containing malignant clones. Sequencing was conducted on the MGISEQ-2000 platform with an Agilent_V6 exome capture kit, generating paired-end 100 bp reads. The WES procedure primarily comprised three key steps: (1) quality control and filtering of raw sequencing data; (2) alignment of the cleaned reads to the human reference genome (GRCh38); and (3) variant calling and annotation, which included the analysis of single nucleotide polymorphisms (SNPs) and insertions/deletions (Indels). Copy number variant (CNV) analysis was not performed on the WES data. The detailed exome sequencing methodology is provided in the Supplementary Material 1. Data from three next-generation sequencing studies indicate that ENKTL and ANKL share common molecular pathways in lymphomagenesis (14). The targeted gene screening panel included potential causative genes from commonly mutated genes in NK-cell origin NK/T-cell lymphoma (*TP53, DDX3X, STAT3, JAK3, MGA, BCOR, ECSIT, MCL1,* and *KMT2D*) and 18 genes associated with hemophagocytic lymphohistiocytosis (*PRF1, UNC13D, STX11, STXBP2, RAB27A, LYST, AP3B1, XIAP, SH2D1A, MAGT1, CD27, ITK, NBAS, NLRC4, CDC42, CD70, CTPS1, RASGRP1*). This panel comprises a subset of known inborn errors of immunity (IEI) genes, as defined by the International Union of Immunological Societies (IUIS). It is important to note that this represents only a fraction of all known IEI genes. Therefore, the possibility of a mutation in a gene not covered by our screening cannot be entirely ruled out. As no paired normal tissue sample was available, the distinction between germline and somatic variants was inferred based on variant allele frequencies (VAFs), which are reported for each variant in Supplementary Table 4. Formal variant classification according to the American College of Medical Genetics and Genomics (ACMG) guidelines was not performed due to the unavailability of parental samples for segregation analysis.

Gene mutations with a mutation frequency greater than 0.01 in all populations and the Asian population in the 1,000 Genomes databases were excluded, resulting in 17 mutations (Supplementary Table 4). Details of all variants of interest are provided in Supplementary Table 4, including chromosomal position, cDNA and protein change, zygosity, VAF, allele frequency in 1,000 Genomes Project and NHLBI-ESP6500 (European American and African American) databases, in silico predictions, and Sanger validation status. A novel heterozygous mutation of *DDX3X* (c.1600C>T;Arg534Cys) on the X-chromosome was detected. This variant has never been found in the 1,000 gESP6500 or Exac databases. It affects a conserved site (GERP++5, 22) and is predicted to be pathogenic by bioinformatic tools, such as Sorting Intolerant From Tolerant (SIFT), PolyPhen2, MetaSVM, MutationTaster, FATHMM, and MetaLR. The Mutation Assessor, VEST3, and CADD algorithms consistently predicted a significant impact on functional changes. Moreover, a missense heterozygous variant of *KMT2D* (c.11954T>A,Leu3985Gln) on chromosome 12 was detected. This variant has never been found in the 1,000 g or ESP6500 databases but has been reported in the Exac database. It affects a conserved site (GERP++4, 85) and was predicted to be pathogenic by SIFT. The Mutation Assessor, VEST3, and CADD algorithms predicted minimal impact on functional changes. Sanger sequencing was performed to confirm the mutation sites in the two genes of interest. Given that the identified heterozygous pathogenic variant in *KMT2D* is a primary cause of Kabuki syndrome type 1 (KS1, MIM: 147920) (15), we retrospectively reviewed the clinical phenotype of the proband. The proband demonstrated Kabuki syndrome-associated features of immunodeficiency and increased susceptibility to respiratory infections; however, other characteristic features such as postnatal growth restriction, distinctive facial features, cardiac abnormalities, skeletal anomalies, abnormal finger pads, and developmental delay/intellectual disability (mild to moderate intellectual impairment) were not observed (16, 17).

## 3 Discussion

The patient presented with a series of complex conditions triggered by EBV infection. Due to the lack of recent medical history, there was no evidence of EBV infection before she developed symptoms of fever, pneumonia, and hepatosplenomegaly. We could not rule out the possibility of CAEBV at the time of admission. Serological testing was performed on day 23 after the onset of symptoms (Supplementary Table 1). The patient's serological results showed the presence of EBV-VCA-IgG and EBV-NA-IgG in the absence of EBV-VCA-IgM, which is typical of past infection (18, 19). EBV-VCA-IgM appears at the onset of clinical symptoms in acute infection and disappears within weeks, and its negativity essentially rules out recently occurred primary acute infection; EBV-VCA-IgG typically emerges with clinical symptoms of acute infection and remains positive for life; EBV-NA-IgG antibodies generally appear 3–4 weeks or a few months after symptom onset and may persist lifelong (18, 20). If this were a primary acute infection, EBV-VCA-IgM would likely be positive and EBV-NA-IgG would likely be negative at 3 weeks after symptom onset (the time of serological testing). The current serological pattern represents a complete conversion from EBV-VCA-IgM positivity to negativity and from EBNA IgG negativity to positivity. We infer that the patient completed the serological switch from the “acute phase” (IgM-positive) to the “convalescent/latent phase” (IgG-positive, IgM-negative) months or even years prior, rather than following the recent symptom onset. However, the presence of high EBV viral load in NK cells and severe clinical symptoms in this context indicate reactivation of latent virus during immunodysregulation, a hallmark of EBV-associated lymphoproliferative disorders such as CAEBV. But the diagnosis of CAEBV was not established due to failure to meet all required diagnostic criteria (such as well-documented duration of symptoms). Therefore, the final diagnosis was confirmed as EBV-HLH and ANKL. CAEBV is primarily seen in pediatric and adolescent patients, with most cases reported in East Asia, and usually with infectious mononucleosis-like symptoms lasting three months or longer (6). CAEBV only includes T-cell or NK-cell diseases (21). The patient in the current study had unexplained high concentrations of IgE (3,590 IU/ml; normal range, 0–100 IU/ml), a feature similar to that of CAEBV patients with NK-cell infection. These patients often present with mild systemic symptoms, high IgE concentrations, relatively low EBV-specific antibody levels, and skin lesions (22, 23). CAEBV disease is a premalignant condition (21), and NK-type CAEBV patients (23.1%) may eventually develop ANKL or ENKTL (23), as in the current case study. ANKL is a systemic NK cell tumor almost always associated with EBV, and HLH is present in less than 50% of the cases (7). ANKL is mainly seen in Asians, mostly in young to middle-aged adults with no definite sex predilection (6). It is characterized by high-grade fever, systemic malaise, hepatosplenomegaly, hepatic failure, and pancytopenia. Lymphadenopathy may occasionally be present, whereas cutaneous manifestations are rare. The disease is frequently complicated by HLH and coagulopathy, demonstrating a fulminant clinical course with progressive multi-organ failure. Some cases arise in the context of CAEBV of NK-cell type or evolve from ENKTL or chronic LPDs of NK cells (6). Although pathological confirmation of ANKL was unavailable in our case, the collective clinical and laboratory findings were highly suggestive of the disease.

The differential diagnosis between ANKL and other malignant diseases within the spectrum of EBV-T/NK-LPDs is challenging. ENKTL may demonstrate clinicopathological features and immunophenotypic characteristics similar to those of ANKL (6). However, the core involvement sites in this patient were the bone marrow, liver, and spleen, accompanied by peripheral blood infiltration, presenting with leukemic dissemination, which is more indicative of ANKL. Additionally, initial examinations revealed the absence of a primary nasal lesion and lymph node involvement, with the disease not primarily manifesting in lymph nodes, thus preliminarily ruling out ENKTL and primary EBV-positive nodal T/NK-cell lymphoma. Combined with the patient's typical NK-cell immunophenotype, this case should be definitively classified as Epstein–Barr virus-associated aggressive natural killer cell leukemia (ANKL).The frequency of CD16 expression is significantly higher in ANKL (75%) than in ENKTL (22%) (14). Although systemic EBV-positive T-cell lymphoma during childhood shares some clinicopathological features with ANKL, the two disease entities can be distinguished by their fundamentally distinct immunophenotypes—CD56^+^ NK cells vs. CD56^−^ T cells. Our patient exhibited features suggestive of HLH prior to progression to ANKL. Inappropriate activation of lymphocytes, monocytes, and macrophages leads to the overproduction of cytokines (24). The classic manifestations of HLH, a rare but fatal hyperinflammatory syndrome, include fever, hepatosplenomegaly, and multiple organ dysfunction syndrome, and is broadly classified into primary (genetic) and secondary forms (infections, autoimmune diseases, malignancies, or immunosuppression) (25). Without treatment, the prognosis is extremely poor, with a median survival time typically under two months (26). HLH can be driven by pure EBV infection, CAEBV, and lymphoma. A 2019 nationwide epidemiological survey of all HLH cases in China revealed EBV as the most common etiology; primary HLH and EBV-associated HLH were predominant in pediatric populations, whereas malignancy-associated HLH was more prevalent in adults (27). The diagnostic challenges of aggressive lymphoma presenting as the initial presentation of HLH due to non-specific symptoms, rapid progress, and inappropriate diagnostic criteria have been reported (28, 29). However, existing reports primarily focus on adults with HLH of unknown etiology, despite receiving HLH-directed therapy, these adult (particularly elderly) patients face a substantial mortality risk, with two reported cases succumbing prior to lymphoma diagnosis. Our case identified a critical knowledge gap: the pediatric presentation of ANKL initially manifesting as EBV-HLH, highlighting unique diagnostic and therapeutic challenges.

The pre-administration of methylprednisolone to the patient for fever and pneumonia initially may have compromised both HLH-specific diagnostic evaluation and recognition of NK-cell lymphoma transformation. Early empirical corticosteroid use should generally be avoided in such cases (30). We adopted reduced perforin expression as a surrogate indicator of potential NK cell functional impairment. Perforin in NK cells serves as a relevant functional marker and a downstream effector linked to both NK cell quantity and activity, and its reduced expression indicates impaired or absent cytolytic function (31, 32). The perforin deficiency is a known mechanism that causes cellular dysfunction in primary HLH. Its expression has been proven to have high accuracy in differentiating patients with primary HLH due to cytotoxicity defects (33, 34). The HLH-2004 diagnostic criteria were established based on the available technology for the diagnostic classification of primary HLH, requiring either a genetic defect or fulfillment of five of eight criteria. However, the specificity and sensitivity of these criteria for secondary HLH remain unclear. They have been pragmatically adopted as the diagnostic criteria for all forms of HLH, which may potentially lead to delayed diagnosis or misdiagnosis (35). Diagnosing multisystem inflammatory diseases requires distinguishing between the classification and diagnostic criteria. Classification criteria are derived from homogeneous cohorts in clinical studies and are highly specific but may exclude some patients. Diagnostic criteria are more sensitive, capturing diverse disease features to identify broader patient populations (36).

A retrospective review of the case revealed that the patient had already met the HLH-2004 criteria at the initial admission to our hospital. However, given the absence of elevated ferritin—a parameter often considered in the diagnostic process—we opted for a conservative management strategy of continued clinical observation, and the diagnosis of HLH was not confirmed at that stage. In contrast to ferritin, the patient's sIL-2R level was already elevated upon initial admission. Although it did not meet the HLH-2004 diagnostic criteria, this finding remained suggestive and highlights the importance of early sIL-2R testing. Of particular interest was the dynamic progression from an initially normal ferritin state to subsequent marked hyperferritinemia accompanied by an sCD25 surge, a temporal evolution fully consistent with the established pathophysiology of HLH. HLH is a life-threatening, systemic hyperinflammatory syndrome associated with increased cytokine production, and the type of anemia in this condition is likely anemia of inflammation, the latter of which features elevated ferritin levels (37). However, when anemia of inflammation coexists with iron deficiency, a pre-existing iron-deficient state may influence changes in biomarker profiles—for example, blunting the expected ferritin elevation (37). We speculate that the cytokine storm triggered by HLH strongly stimulated the production of ferritin as an acute-phase response substance, ultimately reversing the initial iron deficiency and leading to the characteristic hyperferritinemia (38). In our patient, iron deficiency was present at admission, supported by laboratory indices including serum iron (5.08 μmol/L), percent iron saturation (7.93%), unsaturated iron binding capacity (59.02 μmol/L), hemoglobin (103 g/L), and BM cell morphology consistent with iron deficiency. We hypothesize that this may explain why ferritin levels were not elevated in the early stage. As the disease progressed, sCD25 levels gradually increased. sCD25 is a surrogate marker for T cell activation, but it is also released by dendritic cells, activated B cells, monocytes, and malignant cells (39, 40). Its rapid surge indicates a sudden, intense proliferation and activation of the immune system—hallmark features of HLH. In summary, early ferritin levels may reflect a pre-existing condition, whereas the later elevations in hyperferritinemia and sCD25 represent acquired consequences of the widespread inflammatory processes in HLH. This pattern underscores the importance of serial monitoring of these biomarkers and interpreting them within the full clinical context, alongside maintaining a high clinical suspicion for HLH even when classic laboratory criteria are not fully met in the early stages.

Efforts to refine the diagnostic approach for HLH have included the development of novel diagnostic tools. Smits et al. identified a minimal parameter set comprising two major criteria (phagocytosis and splenomegaly) and three minor criteria (cytopenia, increased ferritin, and increased TG/low FIB) for HLH prediction (41). The H-Score, validated for adults and children (42), quantifies HLH probability using the immunosuppression status, fever, organomegaly, TG, ferritin, AST, FIB, cytopenias, and marrow hemophagocytosis (13). Li Xun et al. developed an EBV-HLH screening model for pediatric patients with acute EBV infection, incorporating five routine laboratory parameters: hemoglobin, platelets, neutrophils, albumin, and LDH (43). Subsequently, the team also developed a three-step screening procedure utilizing common clinical and laboratory parameters to effectively identify pediatric patients at high risk for HLH (44). Additionally, recent studies have found that initial laboratory findings, such as ferritin, AST, ALT, LDH, serum sodium, and albumin levels, can aid in identifying suspected early-onset HLH; continuous monitoring is required to detect late-onset HLH in cases of clinical deterioration (45). Clinically, more effective HLH screening methods should be adopted to reduce unnecessary diagnostic tests while minimizing the risk of missed diagnoses, thereby enabling timely intervention and early effective treatment.

The distinction between innate and acquired factors has become increasingly blurred with advances in genetic mutation identification. Many disorders may harbor underlying genetic predispositions, which can lead to disease manifestation through a “second-hit” mechanism upon exposure to external triggers. In the present case, WES identified potential pathogenic variants in the *KMT2D* and *DDX3X* genes. A WES study involving 14 ANKL cases identified mutations in *STAT3* (21%), RAS-MAPK pathway genes (21%), *DDX3X* (29%), and epigenetic modifiers (50%) (46). The epigenetic regulator KMT2D and RNA helicase DDX3X have also been found to be affected by genetic mutations in ENKTCL (7). In CAEBV disease (particularly the NK-cell type), somatic mutations in *DDX3X* and *KMT2D* are considered premalignant lesions that may indicate disease deterioration (7). Moreover, genetic mutations or loss of protein expression in KMT2D are associated with poor survival in ENKTL and may play a role in broader EBV ^+^ T/NK-LPDs such as EBV^+^ TL and ANKL, suggesting an adverse prognostic impact (47). The heterozygous pathogenic variant in *KMT2D* identified in the proband of this study is a known cause of Kabuki syndrome type 1 (15, 48). Kabuki syndrome is a rare multiple malformation disorder characterized by features such as postnatal growth restriction, distinctive facial features, cardiac abnormalities, skeletal anomalies, immunodeficiency, and developmental delay/intellectual disability (mild to moderate intellectual impairment) (16, 17). However, its phenotypic spectrum is highly variable, and not all patients present with the full set of classic features and a phenotypic scoring system has been introduced in clinical practice to aid physicians in making a clinical diagnosis (17, 49). Notably, our proband exhibited a history of recurrent respiratory infections (tonsillitis and pneumonia), which aligns with the immune dysregulation commonly observed in Kabuki syndrome patients, while lacking many of the syndrome's classic hallmarks. This presentation may provide a potential immunological background for the Epstein–Barr virus infection and hemophagocytic lymphohistiocytosis that occurred in this case.

EBV^+^ T/NK-cell LPDs with aberrations in the RIG-I-like receptor (RLR) pathway genes *IFIH1* and/or *DDX3X* exhibited higher EBV viral loads in the plasma and NK cells. In natural killer cell-yielding cells (NKYS), *DDX3X* knockdown not only downregulated RLR signaling activity but also upregulated the expression of EBV-encoded oncogenes (e.g., *LMP1* and *EBNA1*) (11). The interpretation of the *DDX3X* variant identified in this female patient is complex and subject to several limitations. *DDX3X* is located on the X chromosome. In females, who possess two X chromosomes, the phenotypic expression of an X-linked gene variant can be significantly modulated by the process of X-chromosome inactivation (XCI), whereby one X chromosome is randomly silenced in each cell (50, 51). Unfortunately, the XCI pattern in this patient was not determined. A skewed XCI pattern, with a preferential inactivation of the chromosome carrying the wild-type allele, could lead to the expression of the variant allele in the majority of cells and contribute to the disease phenotype (52). Conversely, a random or reverse-skewed pattern would mitigate its effect. Furthermore, without parental DNA for segregation analysis, we cannot definitively determine whether this variant was inherited or occurred *de novo* (a strong indicator of pathogenicity for X-linked disorders). The possibility of mosaicism, while less likely, also cannot be ruled out. Therefore, while the *DDX3X* variant represents a candidate finding, its definitive classification as the primary causative variant awaits further studies, including parental testing and XCI analysis.

The treatment options for T/NK-cell LPD are limited and primarily rely on anecdotal evidence and small studies; no standardized treatment protocol is available to date (53). Allogeneic hematopoietic stem cell transplantation (allo-HSCT) remains the only independent favorable prognostic factor for EBV^+^ T/NK-cell LPDs (11). Nucleoside analogs such as ganciclovir and acyclovir primarily inhibit viral replication and are inefficient in eliminating EBV in chronically infected hosts, as EBV maintains a latent infection state in these tumors (54). Therefore, although EBV plays a critical role in the pathogenesis of this case, ganciclovir or acyclovir have limited efficacy against EBV-T/NK LPD in which EBV remains in a latent state. The treatment of HLH primarily consists of two phases: the first phase follows the standard HLH-94 protocol based on VP-16 (etoposide) and corticosteroids, while the second phase typically involves achieving remission through allo-HSCT. Early administration of VP-16 is critical. Prompt recognition of HLH and initiation of the HLH-94 protocol can reduce early mortality. Treatment delay is an independent adverse prognostic factor in HLH. Although treatment with dexamethasone combined with VP-16 upon HLH diagnosis was initiated in the current case study, the poor outcome indicated delayed therapeutic intervention. However, the resistance and associated mortality of the HLH-94 regimen remain clinically unacceptable (55, 56). The L-DEP regimen (PEG-asparaginase combined with liposomal doxorubicin, etoposide, and methylprednisolone) exhibits clinically meaningful efficacy as salvage therapy for refractory EBV-associated HLH (57–59). Other therapeutic approaches for HLH include gene therapy, adoptive T-cell therapy, and novel cytokine-targeted therapies for suppressing hyper-inflammation (60, 61). Some studies have shown that ruxolitinib and anakinra have potential efficacy as first-line therapeutic option for pediatric HLH (62, 63).

The rarity of ANKL precludes prospective clinical trials, and currently, no consensus exists on an optimal chemotherapy regimen for ANKL management (64). Standard treatment includes L-asparaginase-based chemotherapy followed by allo-HSCT (65). Our patient received an anthracycline-containing chemotherapy regimen (CHOP), which unfortunately represents a “historical” or “suboptimal” approach with poor efficacy. NK tumor cells can produce P-glycoprotein, a known resistance factor for anthracycline-based therapies, contributing to the poor response of ANKL to anthracycline-containing regimens like CHOP (66, 67). Multidrug resistance has led current treatment guidelines to recommend L-asparaginase-based protocols for ANKL, such as SMILE (dexamethasone, methotrexate, ifosfamide, L-asparaginase, etoposide), modified SMILE (dexamethasone, methotrexate, ifosfamide, pegaspargase, etoposide), AspaMetDex (L-asparaginase, methotrexate, dexamethasone), VIDL (etoposide, ifosfamide, dexamethasone, L-asparaginase), DICE (cisplatin, ifosfamide, etoposide, dexamethasone, L-asparaginase), GELOX (gemcitabine, oxaliplatin, L-asparaginase), and P-GEMOX (gemcitabine, oxaliplatin, and peg-asparaginase) (64, 68, 69). The clinical trial NCT03719105 is currently evaluating the efficacy of the modified SMILE (dexamethasone, methotrexate, ifosfamide, pegaspargase, and etoposide) chemotherapy regimen as monotherapy and in combination with pembrolizumab for children, adolescents, and young adults with advanced NK-cell lymphoma and leukemia. Allo-HSCT can provide durable disease control and significantly improve survival outcomes for ANKL patients, particularly those who achieve complete remission prior to transplantation (70, 71). Early identification and referral of ANKL patients for transplant evaluation are crucial, as timely intervention may improve prognosis—delayed allo-HSCT can lead to disease progression and reduced survival benefit, particularly in patients with advanced/recurrent/refractory disease or poor prognostic factors (71, 72). In the current study, the patient was initially scheduled for stem cell transplantation but was unfortunately transferred to another institution. The chemoradiation approaches include concurrent chemoradiotherapy (68), sequential chemoradiotherapy (68), and sandwich chemoradiation (73, 74). For locally aggressive NK/T-cell lymphomas such as limited nasal-type ENKTL, combined chemotherapy and involved-field radiotherapy (IFRT) are standard treatment, with radiotherapy being critical for local disease control and achieving high response rates. However, for systemic ANKL, especially in the leukemic phase, which is inherently a systemic disease with widespread dissemination, the role of radiotherapy is very limited—serving only as palliative treatment for local symptom relief rather than a primary therapeutic modality. Treatment primarily focuses on systemic chemotherapy and transplantation. Emerging ANKL therapies target diverse pathways, including immune checkpoint inhibitors (e.g., PD-1/PD-L1 antibodies), JAK inhibitors (e.g., ruxolitinib), BCL2 inhibitors (e.g., navitoclax, venetoclax), hypomethylating agents (e.g., decitabine), HDAC inhibitors (e.g., vorinostat), aurora kinase inhibitors (e.g., alisertib), heat shock protein (HSP) 90 inhibitors, polo-like kinase inhibitors, cyclin-dependent kinase inhibitors, and polycomb repressive complex 2 (PRC2) modulators (64, 68, 69). Autologous EBV-specific cytotoxic T cells and chimeric antigen receptor T-cell (CAR-T) immunotherapy represent other promising therapeutic approaches for ANKL (75).

This study has several limitations. First, key follow-up data, including survival outcome and whether the patient underwent curative-intent allogeneic hematopoietic stem cell transplantation, are unavailable due to loss to follow-up after transfer. This precludes any definitive conclusions regarding the long-term efficacy of the initial therapy and the ultimate prognosis. We acknowledge the limitations in the pathologic work-up of this case. Definitive confirmatory tests such as EBER-ISH for EBV, immunohistochemistry on a core biopsy, and T-cell receptor clonality assessment were not performed. In the absence of a tissue biopsy, the immunophenotypic profile from flow cytometry, together with cytologic findings, a high NK-cell-specific EBV load, and a clinical picture of HLH and ANKL, constitute strong diagnostic evidence. The absence of EBER-ISH validation due to the lack of representative tumor tissue, which precludes definitive histological confirmation of EBV presence within neoplastic cells. This test would have provided further conclusive evidence of EBV latency within the specific cell population *in situ*. Nevertheless, multiple lines of evidence collectively provide strong support for a central role of EBV in this case. These include the characteristic NK-cell immunophenotype, systemic clinical involvement, markedly elevated peripheral blood EBV DNA load, identification of EBV-infected lymphocyte subpopulations, and consistent serological profiles. A further limitation of this study is the absence of cytotoxic/functional assays for NK cells, such as the CD107a degranulation assay or NK cell killing assay. The unavailability of these assessments was due to clinical urgency and technical constraints during the initial diagnostic workup. These tests would have provided direct evidence of the cytotoxic lymphocyte dysfunction suspected in this case. The genetic analysis in this study has several limitations. Parental segregation analysis remains essential for definitively interpreting the identified genetic variants. The unavailability of parental samples precluded definitive segregation analysis and formal ACMG classification of the identified variants. For the *DDX3X* variant identified in our female patient, the potential impact would further depend on the pattern of X-chromosome inactivation, which could not be assessed. The absence of CNV analysis means that large deletions or duplications could have been missed. Despite these limitations, the WES data provided valuable screening information. To further elucidate the genetic underpinnings of this case, performing WGS, an IEI-specific gene panel and trio-based sequencing would allow for a more unbiased and comprehensive screening. Despite these limitations, the detailed initial presentation, diagnostic work-up, and treatment response documented here provide valuable insights into the management of this rare and aggressive disease.

In summary, T/NK-cell LPDs are exceptionally rare malignancies, and diagnosis is often delayed and challenging due to non-specific clinical presentations. To date, there is no standardized treatment protocol for T/NK-cell LPDs. This case illustrates an instance of ANKL that initially presented with EBV-HLH. Its leukemic dissemination, involvement of the bone marrow core, and characteristic NK-cell phenotype enable its distinction from ENKTL and primary EBV-positive nodal T/NK-cell lymphoma. This case highlights the key clinical aspects of pediatric NK-cell LPD and underscores the pathogenic role of EBV infection in children. Establishing optimized diagnostic workflows and evidence-based therapies for T/NK-cell LPD is imperative to improve patient outcomes.

## 4 Patient perspective

This study related to human use has complied with all the relevant national regulations, institutional policies, and in accordance with the tenets of the Helsinki Declaration, and has been reviewed and approved by the Medical Research Ethics Committee of the Second Hospital of Dalian Medical University. The written informed consent was obtained from her legal representatives included in this study.

## Data Availability

The datasets presented in this article are not readily available due to privacy and ethical concerns regarding the minor patient involved in this case report. Requests to access the datasets should be directed to the corresponding author.
