# Supplementary material for: Hemophagocytic lymphohistiocytosis as the initial manifestation of Epstein–Barr virus-related T/NK-cell lymphoproliferative disorders in a pediatric patient: a case report and literature review
Source: Front Pediatr. 2025 Nov 11;13:1662074. doi: 10.3389/fped.2025.1662074 (PMC12643967; doi:10.3389/fped.2025.1662074)
Supplement: Supplementary file 1 [file Supplementaryfile1.docx]

Supplementary Material 1: Detailed Whole-exome sequencing and Analysis Protocol

1. DNA Sample Preparation

Sample Source: DNA extracted from 2 ml of ethylenediaminetetraacetic acid (EDTA) anticoagulated peripheral whole blood from our patient.

2. Exome Library Preparation

Library Preparation and Sequencing Workflow:

(1) Qualified genomic DNA samples were fragmented by sonication.

(2) DNA fragments of 200-300 bp were selected using magnetic beads.

(3) The selected fragments underwent end repair, followed by a 3'-end adenylation (A-tailing).

(4) Library adapters were ligated to both ends of the DNA fragments.

(5) Adapter-ligated DNA was amplified by linear PCR (LM-PCR) to generate the pre-capture library.

(6) The pre-capture library was hybridized with the exome capture array (Agilent V6) for target enrichment.

(7) Non-specifically bound fragments were washed away.

(8) The captured library was amplified via PCR.

(9) The final library was sequenced on the DNBSEQ platform.

3. Sequencing

Sequencing was conducted on the MGISEQ-2000 platform with an Agilent_V6 exome capture kit, generating paired-end 100 bp reads. The raw image data obtained from sequencing were converted into raw reads (paired-end reads) through base calling using DNBSEQ's proprietary software. These data, stored in the FASTQ file format, are referred to as raw data.

4. Bioinformatic Analysis Pipeline

Bioinformatic analysis began with the raw sequencing data generated from the platform.

4.1 Data Filtering

Raw sequencing data may contain adapter sequences, low-quality bases, and undetermined bases (denoted as 'N'), which can significantly interfere with downstream analysis. Therefore, the raw data first underwent a filtering process to obtain high-quality "clean data" or "clean reads."

To remove sequencing noise, we used SOAPnuke (v2.1.0) [1] (https://github.com/BGI-flexlab/SOAPnuke) with the following filtering steps:

(1) Removal of reads containing adapter sequences.

(2) Discarding of a read pair if the percentage of low-quality bases (Phred quality score < 10) in either read exceeded the default threshold of 50%.

(3) Discarding of a read pair if the percentage of 'N' bases in either read exceeded the default threshold of 10%.

After filtering, the resulting "clean data" was subjected to quality assessment, which included statistics on total read counts, data yield, and quality score distribution.

4.2 Alignment

Next, the clean data from each sample was aligned to the human reference genome (GRCh38/hg38) using the Burrows-Wheeler Aligner (BWA)[2, 3]. Data from each sequencing lane were aligned separately, with a unique read group identifier added to the alignment results. The BWA-MEM algorithm was employed for alignment (BWA, v0.7.17; <http://bio-bwa.sourceforge.net/>). Subsequently, the resulting Sequence Alignment/Map (SAM) files were sorted by genomic coordinate and converted to Binary Alignment/Map (BAM) format using SAMtools (v1.3.1; <http://www.htslib.org/>). During library preparation, PCR amplification can introduce duplicate DNA fragments, leading to the generation of identical sequencing reads, known as PCR duplicates. These duplicates can interfere with variant calling accuracy. Therefore, we used the MarkDuplicates tool from the Genome Analysis Toolkit (GATK) to identify and mark these duplicates, ensuring they are ignored in downstream analyses. The software and command parameters were as follows: GATK MarkDuplicates, v4.1.4.1, <https://gatk.broadinstitute.org/hc/en-us/articles/360037225972-MarkDuplicates-Picard->

4.3 Base Quality Score Recalibration (BQSR)

Base quality score recalibration (BQSR) was performed using known variant sites (e.g., dbSNP, 1000 Genomes Project) to improve the accuracy of base quality scores, thereby enhancing the precision of variant detection. The analysis followed the GATK (Genome Analysis Toolkit) best practices pipeline for variant discovery.

The aligned BAM files were processed using the following GATK [4, 5] tools for duplicate marking and base quality recalibration. First, PCR duplicates were identified and marked. Then, the base quality scores were recalibrated in two steps using the following software:

GATK BaseRecalibrator (v4.1.4.1), https://gatk.broadinstitute.org/hc/en-us/articles/360037593511-BaseRecalibrator

GATK ApplyBQSR (v4.1.4.1), https://gatk.broadinstitute.org/hc/en-us/articles/360037225212-ApplyBQSR

Finally, based on the final alignment results, key quality metrics—including sequencing depth, coverage, and mapping rate for each sample—were calculated and summarized.

4.4 Variant Calling

Both SNPs and InDels were identified simultaneously using the GATK HaplotypeCaller tool. This method operates by performing local de novo assembly of haplotypes in genomic regions with evidence of variation. The initial, raw set of variant calls was stored in VCF format, containing all potential variant sites. The software used was:

GATK HaplotypeCaller, v4.1.4.1, https://gatk.broadinstitute.org/hc/en-us/articles/360037225632-HaplotypeCaller

4.5 Variant Filtering

The raw variant set, containing both SNPs and InDels, required filtering to obtain a high-quality and reliable call set for downstream analysis. A hard-filtering approach was applied for this purpose. First, SNP and InDel calls were separated from the raw VCF file. Then, appropriate filtering parameters were applied to the SNP and InDel sets independently. In the output VCF file, SNPs and InDels labeled as "PASS" constituted the final, high-confidence variant set that passed all filter criteria. The following software tools were used:

GATK SelectVariants, v4.1.4.1, https://gatk.broadinstitute.org/hc/en-us/articles/360037225432-SelectVariants

GATK VariantFiltration, v4.1.4.1, https://gatk.broadinstitute.org/hc/en-us/articles/360037226192-VariantFiltration

For SNP hard-filtering, the adjustable filtering metrics included: QualByDepth (QD), FisherStrand (FS), RMSMappingQuality (MQ), MappingQualityRankSum (MQRankSum), and ReadPosRankSum.

For InDel hard-filtering, the adjustable filtering metrics included: QualByDepth (QD), FisherStrand (FS), and ReadPosRankSum.

Detailed explanations and calculation methods for these filtering parameters can be found in the "Variant Annotations" section on the official GATK website.

4.6 SNP/InDel Annotation

Following the generation of the high-confidence variant set, the variants were annotated using Annodb software. Annodb is a specialized tool for annotating VCF files, integrating multiple databases such as SnpEff, COSMIC, ClinVar, 1000 Genomes Project (1000G), and ESP6500. The annotation process included:

(a) Gene-based annotation: Identifying whether SNPs/InDels resulted in protein-coding changes and amino acid alterations.

(b) Database-based annotation: Determining if variants were present in databases like dbSNP; assessing if their minor allele frequency (MAF) in the 1000 Genomes Project was below 1%; evaluating if the SIFT score for non-synonymous SNPs in coding regions was less than 0.05; checking if the GERP++ conservation score was greater than 2; and retrieving other relevant annotation information.

4.7 Identification of Candidate Variants

Candidate variant prioritization focused on protein-altering mutations (nonsynonymous, splice-site, and frameshift). The filtering pipeline excluded common variants (MAF≥1%) present in the 1000 Genomes Project and NHLBI-ESP6500 (European American and African American) databases. The variants were subjected to in silico pathogenicity prediction using SIFT, PolyPhen-2, Mutation Assessor, Condel, and FATHMM. Variants meeting the deleterious threshold in any of these tools were considered potentially damaging.

References

1. Chen, Y., et al., *SOAPnuke: a MapReduce acceleration-supported software for integrated quality control and preprocessing of high-throughput sequencing data.* GigaScience, 2018. **7**(1).

2. Li H, D.R., *Fast and accurate short read alignment with Burrows-Wheeler transform.* Bioinformatics, 2009. **25**(14): p. 1754–1760.

3. Li, H. and R. Durbin, *Fast and accurate long-read alignment with Burrows–Wheeler transform.* Bioinformatics, 2010. **26**(5): p. 589-595.

4. DePristo, M.A., et al., *A framework for variation discovery and genotyping using next-generation DNA sequencing data.* Nature Genetics, 2011. **43**(5): p. 491-498.

5. McKenna, A., et al., *The Genome Analysis Toolkit: A MapReduce framework for analyzing next-generation DNA sequencing data.* Genome Research, 2010. **20**(9): p. 1297-1303.
