# Supplementary material for: Hemophagocytic lymphohistiocytosis as the initial manifestation of Epstein–Barr virus-related T/NK-cell lymphoproliferative disorders in a pediatric patient: a case report and literature review
Source: Front Pediatr. 2025 Nov 11;13:1662074. doi: 10.3389/fped.2025.1662074 (PMC12643967; doi:10.3389/fped.2025.1662074)
Supplement: Supplementary file 3 [file Table2.docx]

**Supplement**

**Supplementary Table S2. HLH-2004 criteria: Patient Fulfillment Status and Timeline.**

| **HLH-2004 criteria: ≥ 5 must be fulfilled** | **Value** | **Measurement date** | **Met (Y/N)** |
| --- | --- | --- | --- |
| 1.Fever | Peaked at 39°C. | Intermittent fever was present for 15 days prior to the initial admission to our hospital. The fever persisted during the subsequent hospitalization. | Y |
| 2.Splenomegaly | An abdominal ultrasound revealed hepatosplenomegaly. The right oblique diameter of the liver measures 14.4 cm; the spleen measures 6.0 cm in thickness and 19.4 cm in length.  Hepatosplenomegaly: The left lobe of the liver measures 13.7 × 7.6 cm. The right oblique diameter is 14.9 cm, with a subcostal length of 9.2 cm. The spleen measures 6.0 cm in thickness and 18.3 cm in length, with a subcostal span of 9.1 cm. | One week prior to the initial admission to our hospital (November 9).  The initial admission to our hospital (November 15). | Y |
| 3.Cytopenias (affecting ≥ 2 of 3 lineages in the peripheral blood):  1)Hemoglobin <90 g/L  2)Platelets <100x 10^9^/L  3)Neutrophils <1.0x 10^9^/L | Platelets: 77x 10^9^/L  Neutrophils: 0.8x 10^9^/L, Platelets: 73x 10^9^/L | The day before the first admission to our hospital (November 14).  On the first day of the initial admission to our hospital. (November 15). | Y |
| 4.Hypertriglyceridemia and/or hypofibrinogenemia:  1)Fasting triglycerides ≥ 3.0 mmol/L (i.e., ≥ 265 mg/dl)  2)Fibrinogen ≤ 1.5 g/L | Fibrinogen: 1.51 g/L  Fasting triglycerides: 2.95 mmol/L | The day before the first admission to our hospital (November 14).  On the first day of the initial admission to our hospital. (November 15). | Y |
| 5.Hemophagocytosis in bone marrow or spleen or lymph nodes | Examination of the bone marrow (BM) cell morphology indicated pancytopenia with iron deficiency BM and a few histiocytes and hemophagocytic cells.  BM cell morphology examination revealed 1.6% hemophagocytic cells.  The results of the BM cell morphology examination revealed prominent hemophagocytosis and diffuse infiltration by abnormal lymphocytes | On the first day of the initial admission to our hospital. (November 16).  On day 4 of re-admitted to the superior hospital (December 14).  On day 10 of re-admitted to our hospital for the second time (December 28). | Y |
| 6.Low or absent NK-cell activity (according to local laboratory reference) | NK-cell perforin: 68.3% (ref: 81-100) | On the fourth day of the initial admission to our hospital. (November 18). | Y |
| 7.Ferritin ≥ 500 µg/L | Ferritin: 478.2µg/L  Ferritin: 600.09µg/L | On the first day of re-admitted to the superior hospital (December 11).  On the second day of re-admitted to our hospital for the second time (December 20). | Y |
| 8.Soluble CD25 (i.e., soluble IL-2 receptor) ≥ 2400 U/ml | soluble IL-2 receptor: 2134 U/ml  sCD25: 20,404 pg/mL  sCD25: 13405 pg/ml  sCD25: 44560 pg/ml  soluble IL-2 receptor: >7000 U/ml | On the first day of the initial admission to our hospital. (November 16).  On the fourth day of the initial admission to superior hospital. (November 25).  On the 10th day of the initial admitted to the superior hospital (December 1).  At the time of the second discharge from the superior hospital (December 14).  On day 10 of re-admitted to our hospital for the second time (December 28). | Y. Although the level was elevated at the initial admission, it did not meet the diagnostic criteria. It increased significantly upon the second admission. (Due to the discrepancy in units between the results from the superior hospital (pg/ml) and the criteria (U/ml), we are unable to determine whether the criteria are met.) |
